# Synthesis and characterization of new polyamides derived from alanine and valine derivatives

**DOI:** 10.1186/1752-153X-6-128

**Published:** 2012-11-02

**Authors:** Ayman El-Faham, Hammed HAM Hassan, Sherine N Khattab

**Affiliations:** 1 Department of Chemistry, College of Science, King Saud University, P.O. Box 2455, Riyadh 11451, Kingdom of Saudi Arabia; 2 Faculty of Science, Department of Chemistry, Alexandria University, P.O. Box 426, Ibrahimia, Alexandria 21321, Egypt

## Abstract

**Background:**

Many efforts have been recently devoted to design, investigate and synthesize biocompatible, biodegradable polymers for applications in medicine for either the fabrication of biodegradable devices or as drug delivery systems. Many of them consist of condensation of polymers having incorporated peptide linkages susceptible to enzymatic cleavage. Polyamides (PAs) containing α-amino acid residues such as L-leucine, L-alanine and L-phenylalanine have been reported as biodegradable materials. Furthermore, polyamides (PAs) derived from C_10_ and C_14_ dicarboxylic acids and amide-diamines derived from 1,6-hexanediamine or 1,12-dodecanediamine and L-phenylalanine, L-valyl-L-phenylalanine or L-phenylalanyl-L-valine residues have been reported as biocompatible polymers. We have previously described the synthesis and thermal properties of a new type of polyamides-containing amino acids based on eight new symmetric *meta*-oriented protected diamines derived from coupling of amino acids namely; Fomc-glycine, Fmoc-alanine, Fomc-valine and Fomc-leucine with *m*-phenylene diamine or 2,6-diaminopyridine. Results revealed that incorporation of pyridine onto the polymeric backbone of all series decreases the thermal stability.

Here we describe another family of polyamides based on benzene dicarboxylic acid, pyridine dicarboxylic acid, and α-amino acid linked to benzidine and 4,4^′^-oxydianiline to study the effect of the dicarboxylic acid as well as the amino acids on the nature and thermal stability of the polymers.

**Results:**

We report here the preparation of a new type of polyamides based on benzene dicarboxylic acid, pyridine dicarboxylic acid, and α-amino acid linked to benzidine and 4,4^′^-oxydianiline to study the effect of the dicarboxylic acid as well as the amino acids on the nature and thermal stability of polymers. The thermal properties of the polymers were evaluated by different techniques. Results revealed that structure-thermal property correlation based on changing the dicarboxylic acid monomer or the diamine monomer demonstrated an interesting connection between a single change (changing the dicarboxylic acids in each series while the diamine is fixed) and thermal properties. The newly prepared polymers may possess biodegradability and thus may find some applications as novel biomaterials**.**

**Conclusions:**

The thermal properties of the new type of polyamides based on benzene dicarboxylic acid, pyridine dicarboxylic acid, and α-amino acid (alanine and valine) linked to benzidine and 4,4′-oxydianiline were evaluated by thermal gravimetric (TG), differential thermal gravimetric (DTG) and differential thermal analysis (DTA) techniques. Results revealed that the structure-thermal property correlation based on changing the dicarboxylic acid monomer or the diamine monomer demonstrated an interesting connection between a single change (changing the dicarboxylic acids in each series while the diamine is fixed) and thermal properties. In addition, pyridine-containing polymers exhibited semicrystalline characteristic with melting temperature, *T*_*m*_. where none of the valine-containing polymers showed a melting and crystallization peak indicating that the polymers were amorphous. This is expected since L-valine side chain can inhibit close packing and eliminate crystallization. The newly prepared polymers may possess biodegradability and thus may find some applications as novel biomaterials.

## Background

Many efforts have been recently devoted to design, investigate and synthesize biocompatible, biodegradable polymers for applications in medicine for either the fabrication of biodegradable devices or as drug delivery systems [1-5]. Many of them consist of condensation polymers having incorporated peptide linkages susceptible to enzymatic cleavage. Polyamides (PAs) containing α-amino acid residues such as L-leucine, L-alanine and L-phenylalanine have been reported as biodegradable materials [6,7]. Jin *et al*. [8] prepared polyamides, and polyureas containing L-leucine and L-tyrosine residues in the chain. Polyester amides derived from α-amino acids and α-hydroxyacids, the polydepsipeptides, have also been investigated as biodegradable polymers [9,10]. Polyamides (PAs) derived from C_10_ and C_14_ dicarboxylic acids and amide-diamines derived from 1,6-hexanediamine or 1,12-dodecanediamine and L-phenylalanine, L-valyl-L-phenylalanine or L-phenylalanyl-L-valine residues have been reported as a biocompatible polymers [11-13]. Furthermore, an appropriate choice of the number and sequence of the α-amino acids, as well as a balance of hydrophilic and hydrophobic characteristics of the other constituents, makes these polymers susceptible to enzymatic cleavage of the peptide bonds by specific enzymes [6,14-19].

Diamine type monomers derived from glycine [20], (D, L)- and (L)-alanine [21-30], (D, L)- and (L)-phenylalanine [31,32] and various aliphatic diols or from tyrosine–leucine-dipeptide [19,33] and different diamines, were utilized to obtain the polyester amides or polyamides. In general, it is expected that derivatives of L-alanine could be highly crystalline with extensive hydrogen bonding in contrast to the amorphous character of polymers that could be synthesized from α-amino acids with bulky side groups [8,34,35].

We have previously [36] described the synthesis and thermal properties of a new type of polyamides-containing amino acids based on eight new symmetric *meta*-oriented protected diamines derived from coupling of four types of Fmoc-amino acids namely; Fomc-glycine, Fmoc-alanine, Fomc-valine and Fomc-leucine with *m*-phenylene diamine or 2,6-diaminopyridine. Results revealed that incorporation of pyridine onto the polymeric backbone of all series decreases the thermal stability [36].

Here we describe another family of polyamides based of benzene dicarboxylic acid, pyridine dicarboxylic acid, and α-amino acid linked to benzidine and 4,4^′^-oxydianiline to study the effect of the dicarboxylic acid as well as the amino acids on the nature and thermal stability of the polymer.

## Experimental

### Materials and methods

The solvents used were of HPLC reagent grade. The commercial isophthalic acid (Merck), pyridine-2,6-dicarboxylic acid (Aldrich), pyridine-3,5-dicarboxylic acid (Aldrich), Fmoc-amino acids namely Fmoc-Ala-OH **1**, Fmoc-Val-OH **2**, and (*O*-(7-azabenzotriazol-1-yl)-1,1,3,3-tetramethyluronium hexafluorophosphate) (HATU) (IRIS Biotech, Germany), benzidine (Aldrich) **3**, 4,4^′^-oxydianiline (Aldrich) **4** and the solvents triethylamine (Et_3_N), *N,N*-dimethylacetamide (DMAc), *N,N*-dimethylformamide (DMF), 1-Methyl-2-pyrrolidone (NMP) (Fluka), diethylamine, acetonitrile, chloroform, n-hexane, ethyl alcohol were used as purchased without purification.

Melting points were determined with a Mel-Temp apparatus and are uncorrected. Infrared spectra (IR) were recorded on a FTIR-8400S Shimadzu-Japan or on a Perkin-Elmer 1600 series, Fourier transform instrument as KBr pellets. Absorption spectra were measured with a UV 500 UV–vis spectrometer at room temperature (rt) in DMSO with a polymer concentration of 1 mg/10 mL. Magnetic resonance spectra (^1^H NMR and ^13^C NMR spectra) were recorded on a JEOL 500 MHz spectrometer with chemical shift values reported in δ units (ppm) relative to an internal standard. Follow-up of the reactions and checks of the purity of the compounds was done by thin layer chromatography (TLC) on silica gel-protected aluminum sheets (Type 60 GF254, Merck) and the spots were detected by exposure to UV-lamp at λ 254 nm for a few seconds. Differential thermogravimetric (DTG) analyses were carried out in the temperature range from 20°C to 500°C in a steam of nitrogen atmosphere by Shimadzu DTG 60H thermal analyzer. The experimental conditions were: platinum crucible, nitrogen atmosphere with a 30 ml/min flow rate and a heating rate 10°C/min. Differential thermal analysis (TGA/DTA) analyses were carried out using SDT-Q600-V20.5-Build at the Institute of Graduate Studies and Research, Alexandria University and at the Microanalysis Center, Cairo University, Giza, Egypt. Elemental analyses were performed at the Microanalytical Unit, Cairo University and Center for mycology and biotechnology, Alazhar University, Cairo.

### Synthesis of Bis Fmoc-protected diamines 5–8 (general method)

To a solution of Fmoc–Ala-OH **1** (0.623 g, 2 mmol) or Fmoc–Val-OH **2** (0.679 g, 2 mmol); diisopropylethylamine (DIEA, 0.7 mL, 4 mmol) in 5 mL DMF was added HATU (0.76 g, 2 mmol) as a coupling reagent. The reaction mixture was stirred for 3 min (to preactivate the carboxylic acid and form the N-protect amino acid active ester), followed by the addition of a solution of diamine **3** (0.2 g, 1 mmol) or **4** (0.184 g, 1 mmol) in 2 ml DMF. The reaction mixture was stirred overnight and then was poured over water. The precipitate was filtered, washed with 5% citric acid (3 × 20 mL), saturated NaHCO_3_ (3 × 20 mL) and water. The crude product was recrystallized from CH_2_Cl_2_/hexane.

### Bis((9*H*-fluoren-9-yl)methyl) 1,1^′^-(biphenyl-4,4^′^-diylbis(azanediyl))bis(1-oxopropane-2,1-diyl)dicarbamate 5 (FT-IR, ^1^H NMR and ^13^C NMR are attached as supporting information; Additional files 1, 2, 3 respectively)

The reaction of Fmoc-Ala-OH **1** with benzidine **3** gave compound **5**. The product was obtained as a white powder, mp 158-159°C, in yield 0.65 g (84%). IR (KBr): 3301 (NH), 1671 (C=O, amide) cm^-1^. ^1^H-NMR (CDCl_3_, 500Hz): δ 1.23-1.30 (m, 6H, 2 CH_3_), 4.18-4.24 (m, 4H, 4 CH), 6.24 (s, 4H, 2 CH_2_), 6.82, 6.92 (2brs, 2H, 2 NH, D_2_O exchangeable), 7.31-7.86 (m, 24H, Ar-H), 10.11, 10.13 (2s, 2H, 2 NH, D_2_O exchangeable). ^13^C-NMR (CDCl_3_, 125Hz): δ 21.82, 51.41, 110.34, 120.16, 120.54, 121.90, 126.95, 127.90, 129.53, 134.99, 137.88, 138.39, 139.87, 143.00, 154.00, 175.60. *Anal.* Calcd for C_48_H_42_N_4_O_6_: C, 74.79; H, 5.49; N, 7.27. Found: C, 75.02; H, 5.77; N, 6.95.

### Bis((9*H*-fluoren-9-yl)methyl)-1,1^′^-(biphenyl-4,4^′^-diylbis(azanediyl))bis(3-methyl-1-oxobutane-2,1-diyl) dicarbamate 6 (FT-IR, ^1^H NMR and ^13^C NMR are attached as supporting information; Additional files 4, 5, 6 respectively)

The reaction of Fmoc-Val-OH **2** with benzidine **3** gave compound **6**. The product was obtained as a white powder, mp 212-213°C, in yield 0.77 g (93%). IR (KBr): 3289 (NH), 1692, 1660 (C=O, amide) cm^-1^. ^1^H-NMR (CDCl_3_, 500Hz): δ 0.82, 0.89 (2d, 12H, *J*= 6.9 Hz, 4 CH_3_), 1.86-1.90 (m, 2H, 2 CH), 3.08 (d, 2H, *J* = 6.1 Hz, 2 CH), 3.77-3.81 (m, 2H, 2 CH), 6.24 (s, 4H, 2 CH_2_), 7.30, 7.38 (2t, 8H, *J* = 7.6 Hz, Ar-H), 7.51-7.56 (m, 5H, NH+ Ar-H), 7.64-7.69 (m, 5H, NH+ Ar-H), 7.80, 7.84 (2d, 8H, *J* = 7.6 Hz, Ar-H), 9.95, 10.45 (2brs, 2H, 2 NH, D_2_O exchangeable). ^13^C-NMR (CDCl_3_, 125Hz): δ 17.84, 18.99, 19.94, 20.02, 20.15, 61.33, 110.34, 120.08, 120.57, 121.93, 126.96, 127.84, 129.47, 137.94, 139.93, 143.08, 174.50. *Anal.* Calcd for C_52_H_50_N_4_O_6_: C, 75.52; H, 6.09; N, 6.77. Found: C, 75.36; H, 5.95; N, 6.51.

### Bis((9*H*-fluoren-9-yl)methyl)-1,1^′^-(4,4^′^-oxybis(4,1-phenylene)bis(azanediyl))bis(1-oxopropane-2,1-diyl)dicarbamate 7 (FT-IR, ^1^H NMR and ^13^C NMR are attached as supporting information; Additional files 7, 8, 9 respectively)

The reaction of Fmoc-Ala-OH **1** with 4,4′-oxydianiline **4** gave compound **7**. The product was obtained as a white powder, mp 133-134°C, in yield 0.66 g (84%). IR (KBr): 3452, 3288 (NH), 1668 (C=O, amide) cm^-1^. ^1^H-NMR (CDCl_3_, 500Hz): δ 1.27 (d, 6H, 2 CH_3_), 4.18-4.24 (m, 8H, 4 CH+ 2 CH_2_), 6.90 (d, 4H, Ar-H), 7.27-7.32 (m, 4H, Ar-H), 7.38 (t, 4H, Ar-H), 7.56 (d, 4H, Ar-H), 7.65 (d, 1H, NH, D_2_O exchangeable), 7.71 (t, 4H, Ar-H), 7.80 (d, 1H, NH, D_2_O exchangeable), 7.85 (d, 4H, Ar-H), 9.99 (s, 2H, 2 NH, D_2_O exchangeable). ^13^C-NMR (CDCl_3_, 125Hz): δ 21.66, 51.17, 110.31, 119.22, 120.50, 121.67, 121.87, 127.94, 129.57, 134.63, 137.83, 139.82, 142.93, 153.15, 175.46. *Anal.* Calcd for C_48_H_42_N_4_O_7_: C, 73.27; H, 5.38; N, 7.12. Found: C, 73.64; H, 5.09; N, 6.87.

### Bis((9*H*-fluoren-9-yl)methyl)-1,1^′^-(4,4^′^-oxybis(4,1-phenylene)bis(azanediyl))bis(3-methyl-1-oxobutane-2,1-diyl)dicarbamate 8 (FT-IR, ^1^H NMR and ^13^C NMR are attached as supporting information; Additional files 10, 11, 12 respectively)

The reaction of Fmoc-Val-OH **2** with 4,4′-oxydianiline **4** gave compound **8**. The product was obtained as a white powder, mp 193-194°C, in yield 0.75 g (89%). IR (KBr): 3292 (NH), 1691, 1660 (C=O, amide) cm^-1^. ^1^H-NMR (CDCl_3_, 500Hz): δ 0.80, 0.87 (2d, 12H, *J*= 6.9 Hz, 4 CH_3_), 1.84-1.90 (m, 2H, 2 CH), 3.04 (d, 2H, *J* = 5.4 Hz, 2 CH), 6.24 (s, 4H, 2 CH_2_), 6.90 (d, 4H, *J*= 8.4 Hz, Ar-H), 7.30, 7.38 (2t, 8H, *J*= 7.6 Hz, Ar-H), 7.50-7.58 (m, 2H, 2 NH, D_2_O exchangeable), 7.60 (d, 4H, *J*= 8.4 Hz, Ar-H), 7.80, 7.84 (2d, 8H, *J*= 7.6 Hz, Ar-H), 9.83, 10.47 (2brs, 2H, 2 NH, D_2_O exchangeable). ^13^C-NMR (CDCl_3_, 125Hz): δ 17.85, 20.16, 31.10, 61.27, 110.36, 119.18, 120.57, 121.34, 121.93, 127.83, 129.46, 134.98, 137.94, 139.94, 143.08, 152.99, 174.29. *Anal.* Calcd for C_52_H_50_N_4_O_7_: C, 74.09; H, 5.98; N, 6.65. Found: C, 73.87; H, 5.53; N, 6.21.

### General procedure for the deblocking of the Fmoc-protecting groups: preparation of the diamines 9–12

Protected diamine (0.5 mmol) **5**–**8** was stirred with 40 ml (30% Et_2_NH / CH_3_CN) at r.t. for 14h. The progress of the reaction was monitored by using TLC using ethyl acetate / hexane 4:6 *v/v* as eluent. The solvent and volatiles were removed under reduced pressure and the crude residue was washed thoroughly with hexane to get rid from the deblocked dibenzofulvene byproduct to produce the desired diamine **9**–**12** which is used directly to the next step.

### Preparation of polymers 16–25 by low-temperature solution polycondensation (general method)

To a mechanically stirred cold (ice bath) solution of the diamine **9**–**12** (1.0 mmol) dissolved in 5.0 mL DMA, a solution of 1.0 mmol of the acid dichloride **13**, **14, 15** dissolved in 5.0 mL DMA was added dropwise. The reaction mixture was allowed to stir for 2h then the mixture was poured into iced water. The formed polymer precipitate was filtered under vacuum, washed thoroughly with water, ethyl alcohol and water again, dried and kept in the desiccator.

### Poly[3-acetyl-*N*-((2S)-1-(4^′^-(2-(methylamino)propanamido)biphenyl-4-ylamino)-1-oxopropan-2-yl)benzamide] 16

The polymerization of the diamine **9** with isophthaloyl dichloride **13** produced the polymer **16** as a black solid, yield 59.0%, m. p. > 300°C, UV (DMSO): λmax= 272 nm (ε =1569), λmax= 304 nm (ε =1684), IR (cm^-1^): 3230 (N-H, amide), 3064 (=C–H, aromatic), 2921 (C–H, aliphatic), 1658 (C=O, amide), 1599 and 1497 (C=C, aromatic), 1114 and 1071 (C–N, aliphatic). Calculated for C_26_H_26_N_4_O_5_; C, 65.81; H, 5.52; N, 11.81; Found: C, 65.52; H, 5.78; N, 11.55.

### Poly[3-acetyl-*N*-((2S)-3-methyl-1-(4^′^-(3-methyl-2-(methylamino) butanamido)biphenyl-4-ylamino)-1-oxobutan-2-yl)benzamide] 17

The polymerization of the diamine **10** with isophthaloyl dichloride **13** produced the polymer **17** as a black solid, yield 56.0%, m. p. > 300°C, UV (DMSO): λmax= 275 nm (ε =2244), λmax= 300 nm (ε =2156), IR (cm^-1^): 3430 (N-H, amide), 3039 (=C–H, aromatic), 2921 (C–H, aliphatic), 1658 (C=O, amide), 1607 and 1446 (C=C, aromatic), 1249 and 1113 (C–N, aliphatic). Calculated for C_30_H_34_N_4_O_5_; C, 67.91; H, 6.46; N, 10.56; Found: C, 67.72; H, 6.70; N, 10.77.

### Poly[3-acetyl-*N*-((2S)-1-(4-(4-(2-(methylamino)propanamido)phenoxy) phenylamino)-1-oxopropan-2-yl)benzamide] 18

The polymerization of the diamine **11** with isophthaloyl dichloride **13** produced the polymer **18** as a black solid, yield 68.0%, m. p. > 300°C, UV (DMSO): λmax= 268 nm (ε =1359), λmax= 276 nm (ε =1169), λmax= 362 nm (ε = 86), IR (cm^-1^): 3439 (N-H, amide), 3096 (=C–H, aromatic), 2935 (C–H, aliphatic), 1669 (C=O, amide), 1592 and 1490 (C=C, aromatic), 1114 and 1070 (C–N , aliphatic). Calculated for C_26_H_26_N_4_O_6_; C, 63.66; H, 5.34; N, 11.42; Found: C, 63.29; H, 5.08; N, 11.73.

### Poly[3-acetyl-*N*-((2S)-3-methyl-1-(4-(4-(3-methyl-2-(methylamino)butanamido) phenoxy)phenylamino)-1-oxobutan-2-yl)benzamide] 19

The polymerization of the diamine **12** with isophthaloyl dichloride **13** produced the polymer **19** as a black solid, yield 60.0%, m. p. > 300°C, UV (DMSO): λmax= 268 nm (ε =1754), λmax= 330 nm (ε =158), IR (cm^-1^): 3477 (N-H, amide), 3036 (=C–H, aromatic), 2887 (C–H, aliphatic), 1640 (C=O, amide), 1610 and 1475 (C=C, aromatic), 1216 and 1150 (C–N, aliphatic). Calculated for C_30_H_34_N_4_O_6_; C, 65.92; H, 6.27; N, 10.25; Found: C, 66.28; H, 6.61; N, 10.60.

### Poly[6-acetyl-*N*-((2S)-1-(4^′^-(2-(methylamino)propanamido)biphenyl-4-ylamino)-1-oxo propan-2-yl)picolinamide] 20

The polymerization of the diamine **9** with pyridine-2,6-dicarbonyl dichloride **14** produced the polymer **20** as a black solid, yield 56.0%, m. p. > 300°C, UV (DMSO): λmax= 268 nm (ε =1930), λmax= 321 nm (ε =459), IR (cm^-1^): 3400 (N-H, amide), 3063 (=C–H, aromatic), 2934 (C–H, aliphatic), 1668 (C=O, amide), 1590 and 1489 (C=C, aromatic), 1440 (C–N , aromatic), 1113 and 1070 (C–N, aliphatic). Calculated for C_25_H_25_N_5_O_5_; C, 63.15; H, 5.30; N, 14.73; Found: C, 63.41; H, 5.06; N, 14.61.

### Poly[6-acetyl-*N*-((2S)-3-methyl-1-(4^′^-(3-methyl-2-(methylamino) butanamido)biphenyl-4-ylamino)-1-oxobutan-2-yl)picolinamide] 21

The polymerization of the diamine **10** with pyridine-2,6-dicarbonyl dichloride **14** produced the polymer **21** as a black solid, yield 62.0%, m. p. > 300°C, UV (DMSO): λmax= 275 nm (ε = 2162), λmax= 299 nm (ε =2106), IR (cm^-1^): 3416 (N-H, amide), 3039 (=C–H, aromatic), 2890 (C–H, aliphatic), 1642 (C=O, amide), 1613 and 1477 (C=C, aromatic), 1445 (C–N , aromatic), 1215 and 1150 (C–N , aliphatic). Calculated for C_29_H_33_N_5_O_5_; C, 65.52; H, 6.26; N, 13.17 Found: C, 65.85; H, 5.96; N, 12.89.

### Poly[6-acetyl-*N*-((2S)-1-(4-(4-(2-(methylamino)propanamido)phenoxy) phenylamino)-1-oxopropan-2-yl)picolinamide] 22

The polymerization of the diamine **11** with pyridine-2,6-dicarbonyl dichloride **14** produced the polymer **22** as a black solid, yield 58.0%, m. p. > 300°C, UV (DMSO): λmax= 268 nm (ε = 767), λmax= 276 nm (ε =651), IR (cm^-1^): 3440 (N-H, amide), 3096 (=C–H, aromatic), 2936 (C–H, aliphatic), 1692 (C=O, amide), 1591 and 1490 (C=C, aromatic), 1441 (C–N , aromatic), 1113 and 1070 (C–N , aliphatic). Calculated for C_25_H_25_N_5_O_6_; C, 61.09; H, 5.13; N, 14.25 Found: C, 61.32; H, 5.44; N, 13.98.

### Poly[5-acetyl-*N*-((2S)-1-(4^′^-(2-(methylamino)propanamido)biphenyl-4-ylamino)-1-oxopropan-2-yl)nicotinamide] 23

The polymerization of the diamine **9** with pyridine-3,5-dicarbonyl dichloride **15** produced the polymer **23** as a black solid, yield 67.0%, m. p. > 300°C, UV (DMSO): λmax= 268 nm (ε =957), λmax= 276 nm (ε =860), λmax= 322 nm (ε =179), IR (cm^-1^): 3416 (N-H, amide), 3087 (=C–H, aromatic), 2936 (C–H, aliphatic), 1669 (C=O, amide), 1593 and 1490 (C=C, aromatic), 1441 (C–N , aromatic), 1113 and 1070 (C–N, aliphatic). Calculated for C_25_H_25_N_5_O_5_; C, 63.15; H, 5.30; N, 14.73; Found: C, 63.33; H, 5.66; N, 14.99.

### Poly[5-acetyl-*N*-((2S)-3-methyl-1-(4^′^-(3-methyl-2-(methylamino) butanamido)biphenyl-4-ylamino)-1-oxobutan-2-yl)nicotinamide] 24

The polymerization of the diamine **10** with pyridine-3,5-dicarbonyl dichloride **15** produced the polymer **24** as a black solid, yield 58.0%, m. p. > 300°C, UV (DMSO): λmax= 269 nm (ε =888), λmax= 276 nm (ε =731), IR (cm^-1^): 3420 (N-H, amide), 3036 (=C–H, aromatic), 2881 (C–H, aliphatic), 1639 (C=O, amide), 1612 and 1475 (C=C, aromatic), 1444 (C–N , aromatic), 1215 and 1150 (C–N , aliphatic). Calculated for C_29_H_33_N_5_O_5_; C, 65.52; H, 6.26; N, 13.17 Found: C, 65.17; H, 6.54; N, 13.51.

### Poly[5-acetyl-*N*-((2S)-1-(4-(4-(2-(methylamino)propanamido)phenoxy) phenylamino)-1-oxopropan-2-yl)nicotinamide] 25

The polymerization of the diamine **11** with pyridine-3,5-dicarbonyl dichloride **15** produced the polymer **25** as a black solid, yield 54.0%, m. p. > 300°C, UV (DMSO): λmax= 269 nm (ε =1871), λmax= 367 nm (ε =166), IR (cm^-1^): 3440 (N-H, amide), 3085 (=C–H, aromatic), 2934 (C–H, aliphatic), 1668 (C=O, amide), 1591 and 1489 (C=C, aromatic), 1440 (C–N , aromatic), 1113 and 1070 (C–N , aliphatic). Calculated for C_25_H_25_N_5_O_6_; C, 61.09; H, 5.13; N, 14.25 Found: C, 60.98; H, 4.96; N, 14.63.

## Results and discussion

### Chemical preparation of the polyamides containing amino acids

#### Preparation of the Fmoc-protected diamines 5–8

**Scheme 1:**
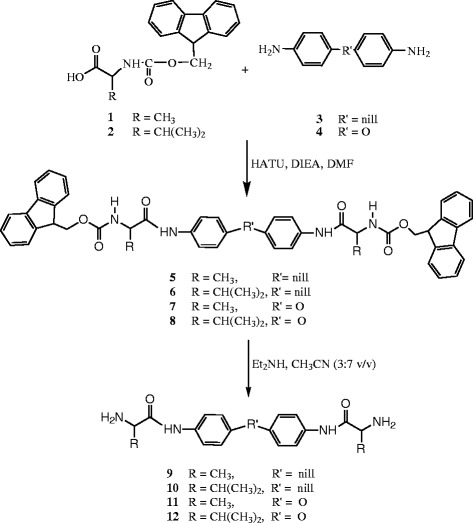
Preparation of the diamines-containing amino acids 9–12.

The preparation of the new symmetric diamines **9–12**, Scheme 1, for the stepwise polymerization was our first target. Fmoc-alanine (Fmoc-Ala-OH) **1** and Fmoc-valine (Fmoc-Val-OH) **2** were used in this investigation. Reactions of two equivalent amounts of the aforementioned amino acids with benzidine **3**, or 4,4^′^-oxydianiline **4** were performed using two equivalents of HATU [37] as coupling reagent in presence of four equivalent of diisopropylethyl amine (DIEA) as base in dimethylformamide (DMF) to furnish the corresponding bis-Fmoc-protected diamines (**5–8**) in good yield and purity. The structures of the prepared protected diamines **5–8** were fully characterized by IR, ^1^H-NMR, ^13^C-NMR and elemental analyses. IR spectra of the protected diamines exhibited characteristic absorption bands in the range 3452–3288 cm^−1^ corresponding to the N-H bond. In addition the bands corresponding to the amide CONH group is observed in the range 1692–1668 cm^−1^. ^1^H-NMR spectra of compounds **58** in DMSO-d_6_ showed signals corresponding to four NH protons. The signals at the range δ 6.82-7.80 ppm correspond to two NH protons, and the other two NH protons are observed at the range δ 9.83-10.47 ppm. The 24 aromatic protons of compounds **5** and **6** are observed at the range δ 7.30-7.86 ppm, while those of compounds **7** and **8** are observed at the range 6.90-7.84 ppm. The ^13^C-NMR spectra of compounds **58** in DMSO-d_6_ show two signals corresponding to the four carbonyl groups at the range 152.99-154.00 ppm and at the range 174.29-175.60.

### Preparation of the diamines-containing amino acids 9–12

Treatment of the protected diamines **5**–**8** with (3:7 Et_2_NH/CH_3_CN *v*/*v*) easily furnished the required diamines **9–12**, respectively, in high yield, Scheme 1. Noteworthy, the byproduct dibenzofulvene was easily removed from the crude materials by washing with *n-*hexane. The crude products obtained were used as such without further purification.

### Preparation of the polyamides 16–25 by low temperature solution polycondensation

**Scheme 2:**
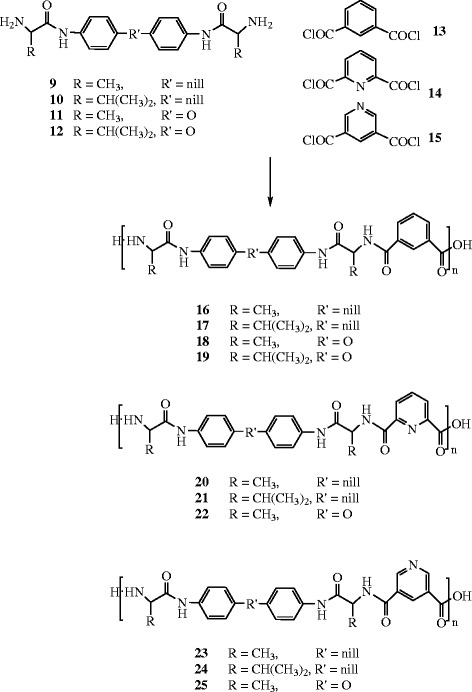
Preparation of the polyamides 16–25 by low temperature solution polycondensation.

The aromatic acid chlorides, namely isophthaloyl dichloride **13,** pyridine-2,6-dicarbonyl dichloride **14**, and pyridine-3,5-dicarbonyl dichloride **15** used in this investigation were prepared by the reaction of their corresponding dicarboxylic acids, isophthalic acid, pyridine-2,6-dicarboxylic acid, and pyridine-3,5-dicarboxylic acid respectively, with thionyl chloride in the presence of few drops of DMF. Direct polycondensation reaction of an equimolar mixture of the acid chloride **13** with the diamines **9**–**12** in DMAc solution at 0–5°C furnished the corresponding polyamides containing amino acids **16–19**, respectively in high yields, Scheme 2. In a similar manner, reactions of the acid chlorides **14** and **15** with the diamines-containing amino acids **9–11** furnished the corresponding polyamides **20–22** and **23–25**, Scheme 2. The polymer structures were confirmed by elemental analysis, IR and UV spectroscopy.

### Physical properties of the prepared polyamides containing amino acids

#### Solubility

The prepared polymers **16–25** showed similar solubility behavior in different organic solvents. Moderate to complete dissolution was observed in a variety of aprotic solvents such as NMP, DMSO, DMAc, boiling alcoholic solvents such as methanol, ethanol while insoluble in halogenated solvents such as CHCl_3_, CCl_4_, CH_2_Cl_2_, ClCH_2_CH_2_Cl or in ethers such as Et_2_O, THF, 1,4-dioxane or 1,2-dimethoxyethane (DME).

### FTIR Spectroscopy

The FTIR spectra of the polymers exhibited characteristic absorbance at the range of *υ* 3477*–*3230 cm^−1^ and 1692–1639 cm^−1^, corresponding to the N-H and C = O stretching of the amide group, respectively. Bands around *υ* 2900 cm^−1^ were assigned to the alkyl H-C stretching, while bands appeared around *υ* 3050, 1598 and *υ* 1524 cm^−1^ assigned to the aromatic C-H and C=C aromatic, respectively.

### Optical properties

The optical properties of polymers **16–25** were investigated by UV–vis spectroscopy in DMSO with a polymer concentration of 1 mg/10 mL. The spectra were recorded from 600 nm to 200 nm and the maximum absorbances (λmax) of the prepared polymers were recorded. Ala-containing polymers **18**, **20, 23** and **25** exhibited bathochromic or red shifted peaks maxima at *λ* 362 nm, 321 nm, 322 nm and 367 nm, respectively may be attributed to the n→*π* transition while peaks at lower wavelengths appeared, respectively at 276 nm, 268 nm, 276 nm and 269 nm and could be attributed to the *π*→*π* transitions. **18** and **23** showed additional peak maxima at 268 nm, due to *π*→*π* transitions. Ala-containing polymers **16** and **22** exhibited peaks at wavelengths, appeared respectively at 304 nm and 276 nm which could be attributed to the *π*→*π* transitions. They also showed additional peak maxima at 272 nm and 268 nm due to *π*→*π* transitions.

Val-containing polymers **17**, **19**, **21** and **24** showed redshifted peaks maxima at *λ* 300 nm, 330 nm, 299 nm and 276 nm corresponding to the expected n→*π*, and similar peaks maxima at *λ* 275 nm, 268 nm, 275 nm and 269 nm, respectively due to *π*→*π* transitions.

### Thermal properties

The thermal properties of these new materials were carried out in the temperature range from 20°C to 500°C in a stream of nitrogen atmosphere. Because many of the polymers containing amino acids were rather hydrophilic and could absorb atmospheric moisture during preparations, samples were heated to remove the absorbed water, cooled, and reheated again at a heating/cooling rate of 20°C/min.

**Figure 1:**
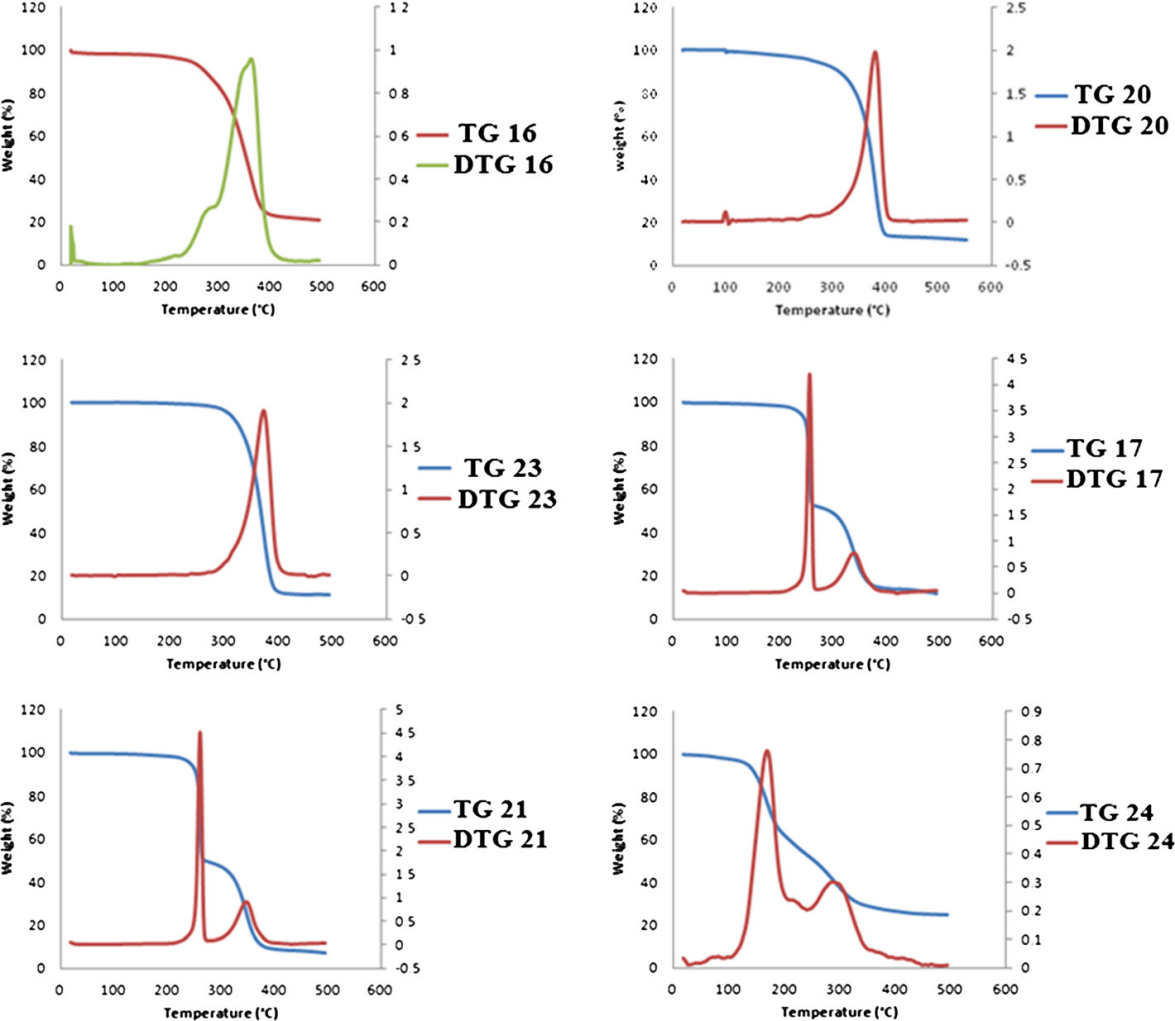
TGA/DTG curves of the alanine-containing polymers 16, 20, 23 and valine-containing polymers 17, 21, 24.

**Figure 2:**
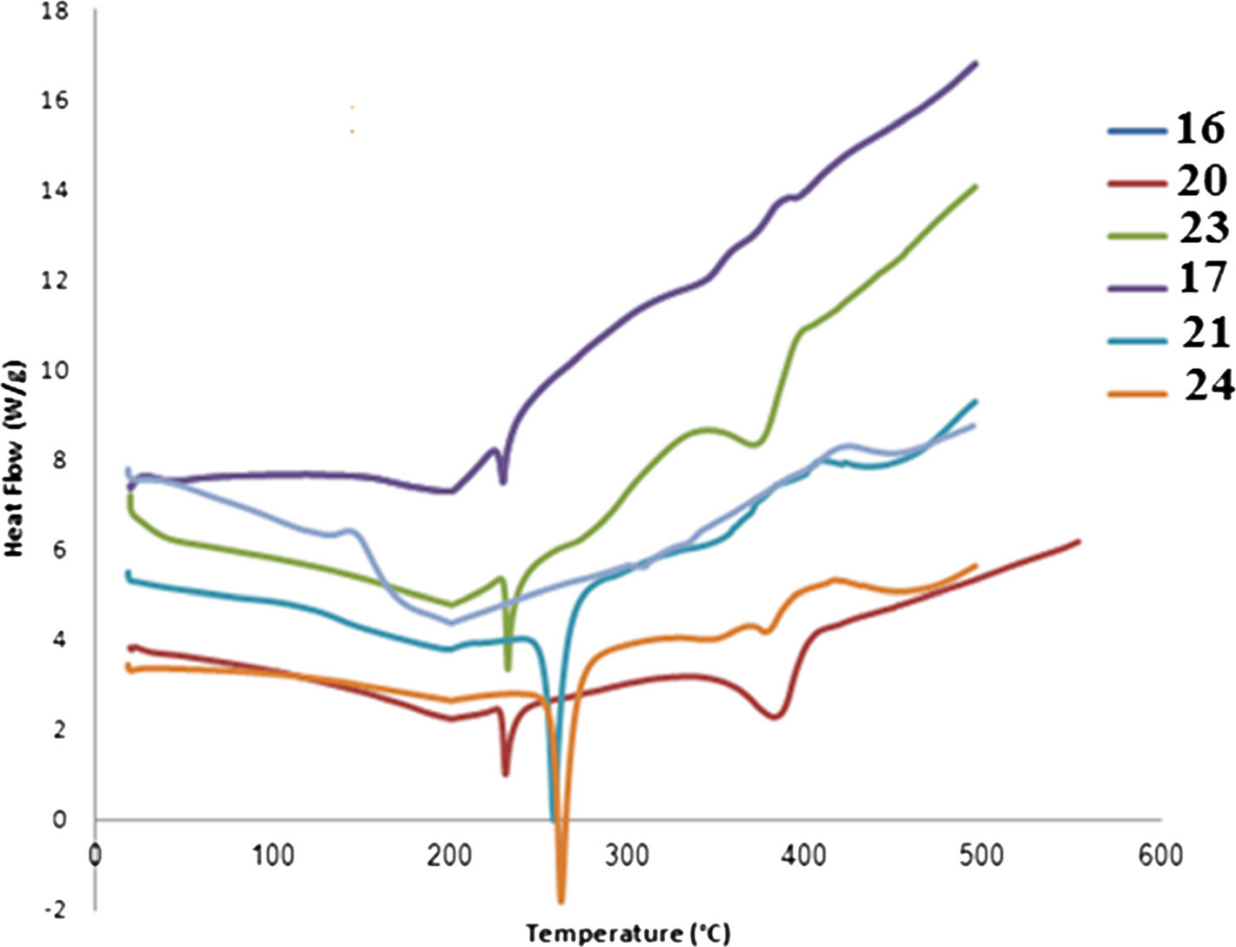
DSC curves of the alanine-containing polymers 16, 20, 23 and valine-containing polymers 17, 21, 24.

Figures 1 and 2 show the TGA/DTG and DSC curves of the alanine-containing polymers **16, 20, 23** and valine-containing polymers **17, 21, 24**, derived from the diamine **3**, respectively. Structure-thermal property correlation based on changing the diacid residue revealed that the prepared polymers have comparable thermal stabilities. Alanine-containing polymers **16, 20, 23** exhibited subsequent degradation and their major amide linkage degradation processes appeared at 365°C (77.12% wt loss), 380°C (81.94% wt loss) and 375°C (86.74% wt loss) leaving 20.69%, 12.64% and 11.39%, respectively, as remaining mass residues. Valine-containing polymers **17, 21, 24** exhibited two subsequent major degradation processes appeared at [256°C (46.91% wt loss), 339°C (37.71% wt loss)] and [260°C (48.77% wt loss), 340°C (39.70% wt loss)] and [168°C (38.00% wt loss), 293°C (35.09% wt loss)] leaving 11.70%, 7.18% and 24.83%, respectively, as remaining mass residues. On the other hand, alanine-containing polymers **22** and **25** derived from the diamine **4** exhibited subsequent degradation processes appeared in the temperature ranges 219°C – 439°C (58.99% wt loss) and 300°C - 495°C (50.53% wt loss) leaving 19.69% and 37.57%, respectively, as remaining mass residues.

The glass transition temperature, *T*_*g*_, of the newly synthesized polymers ranged from 200°C to 225°C, and most of them were amorphous. In case of alanine-containing polymers **16**, **20**, **23**, it is interesting to note that the change of the diacid had a noticeable *T*g difference. In addition, pyridine-containing polymers **20** and **23** exhibited semicrystalline characteristic with melting temperature, *T*_*m*_, 383°C and 378°C, respectively. None of the valine-containing polymers **17**, **21**, **24** showed a melting and crystallization peak indicating that the polymers were amorphous. This is expected since L-valine side chain can inhibit close packing and eliminate crystallization.

## Conclusions

The Thermal properties of the new types of polyamides based on benzene dicarboxylic acid, pyridine dicarboxylic acid, and α-amino acid (Alanine and Valine ) linked to benzidine and 4,4^′^-oxydianiline were evaluated by thermal gravimetric (TG), differential thermal gravimetric (DTG) and differential thermal analysis (DTA) techniques. Results revealed that structure-thermal property correlation based on changing the dicarboxylic acid monomer or the diamine monomer demonstrated an interesting connection between a single change (changing the dicarboxylic acids in each series while the diamine is fixed) and thermal properties. In addition, pyridine-containing polymers exhibited semicrystalline characteristic with melting temperature, *T*_*m*_. where none of the valine-containing polymers showed a melting and crystallization peak indicating that the polymers were amorphous. This is expected since L-valine side chain can inhibit close packing and eliminate crystallization. The newly prepared polymers may possess biodegradability and thus may find some applications as novel biomaterials.

## Competing interests

The authors declare that they have no competing interests.

## Authors’ contributions

HAMH carried out the polymerization, SNK carried out the preparation of the monomers. AEF, HAMH and SNK designed the proposed methods and analyzed the data statistically together. All authors read and approved the final manuscript.

## Supplementary Material

Additional file 1 FT-IR spectra of compound 5. Click here for file

Additional file 2 1H NMR spectra of compound of compound 5. Click here for file

Additional file 3 13C NMR spectra of compound of compound 5. Click here for file

Additional file 4 FT-IR spectra of compound 6. Click here for file

Additional file 5 1H NMR spectra of compound of compound 6. Click here for file

Additional file 6 13C NMR spectra of compound of compound 6. Click here for file

Additional file 7 FT-IR spectra of compound 7. Click here for file

Additional file 8 1H NMR spectra of compound of compound 7. Click here for file

Additional file 9 13C NMR spectra of compound of compound 7. Click here for file

Additional file 10 FT-IR of compound 8. Click here for file

Additional file 11 1H NMR spectra of compound of compound 8. Click here for file

Additional file 12 13C NMR spectra of compound of compound 8. Click here for file
