# Supplementary figures and images for: Synthesis and characterization of new polyamides derived from alanine and valine derivatives
Source: Chem Cent J. 2012 Nov 2;6:128. doi: 10.1186/1752-153X-6-128 (PMC3537673; doi:10.1186/1752-153X-6-128)

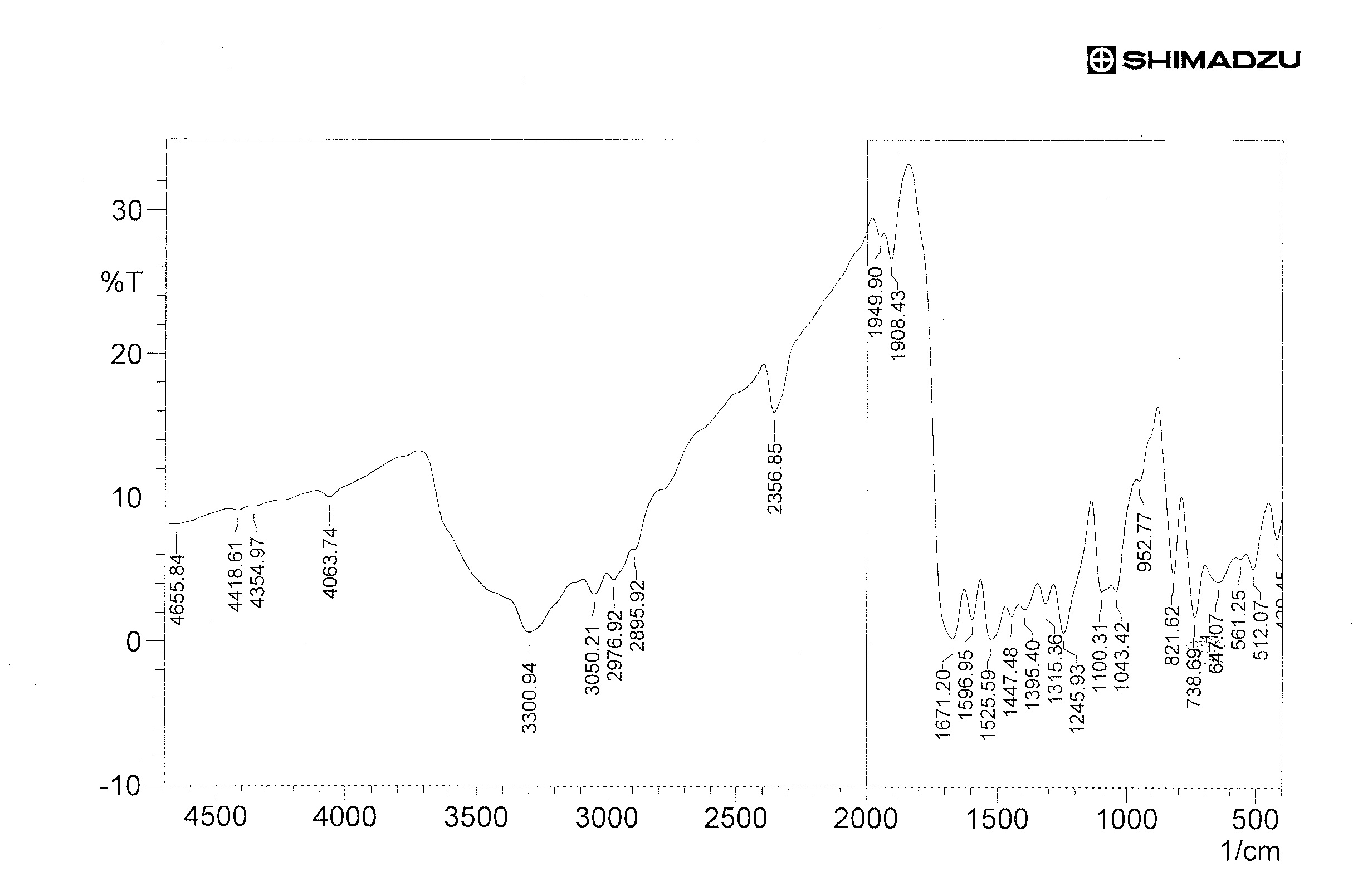

Supplement: Additional file 1 — FT-IR spectra of compound 5. [file 1752-153X-6-128-S1.jpeg]

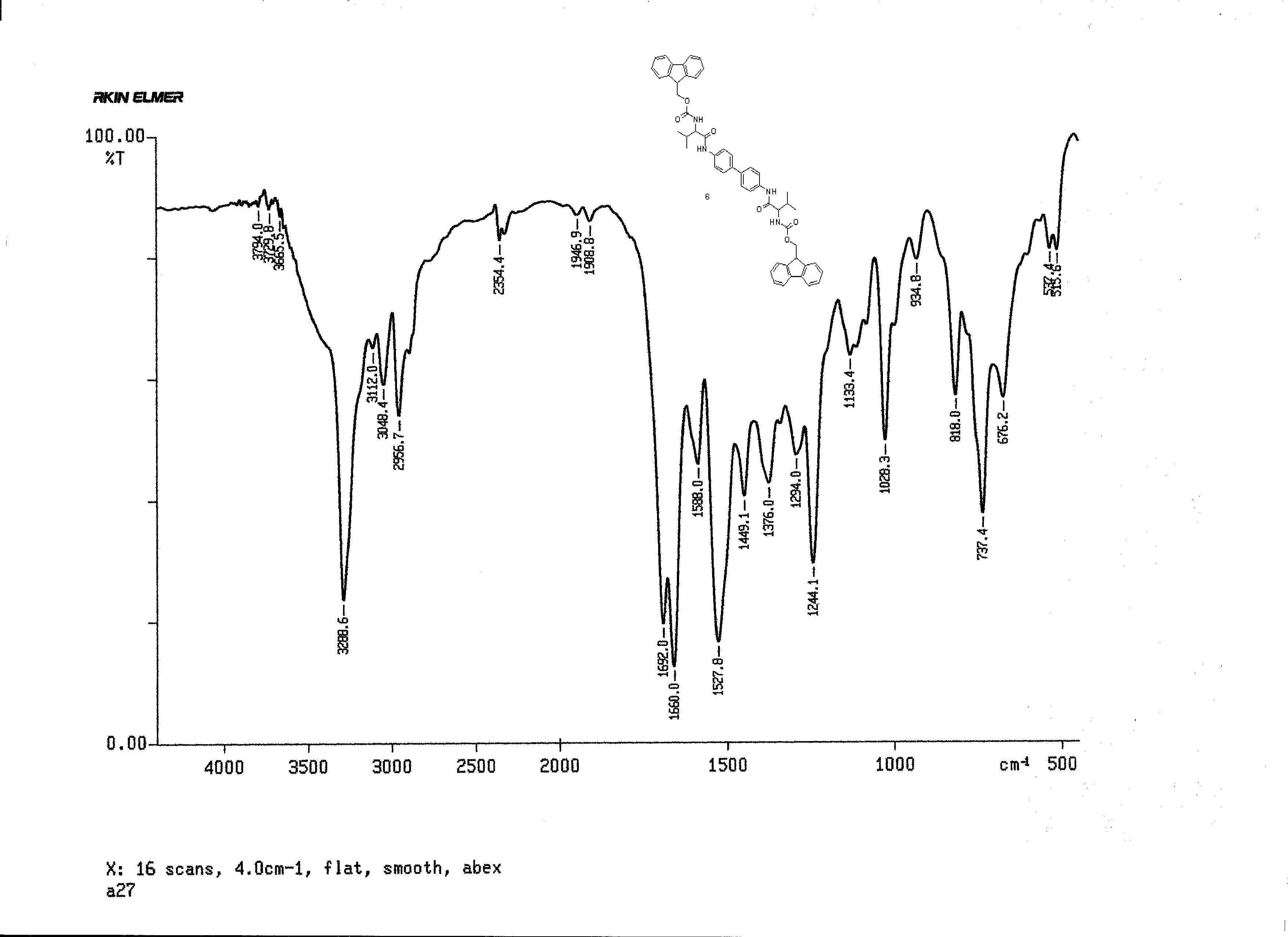

Supplement: Additional file 2 — 1H NMR spectra of compound of compound 5. [file 1752-153X-6-128-S2.tiff]

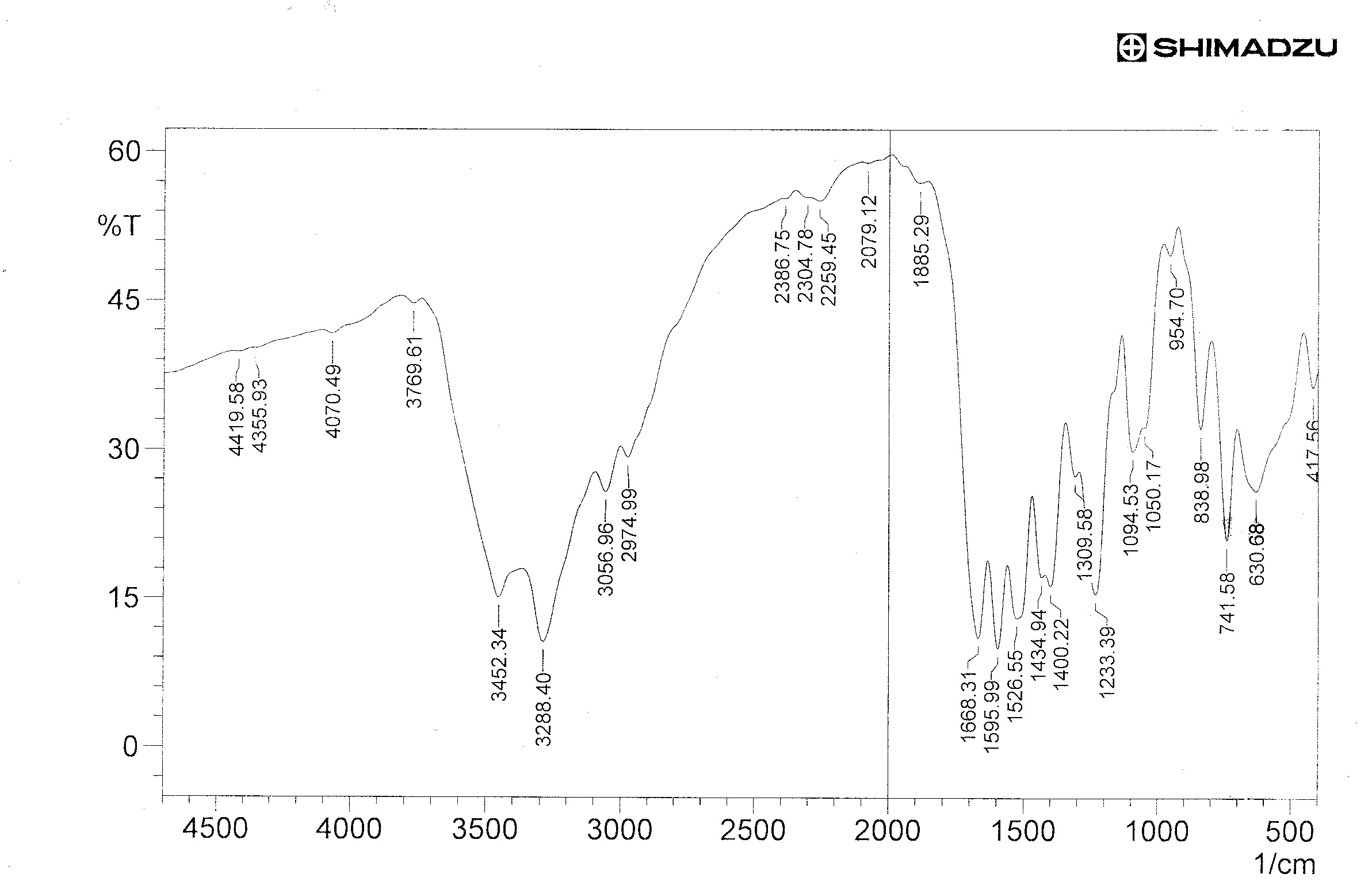

Supplement: Additional file 3 — 13C NMR spectra of compound of compound 5. [file 1752-153X-6-128-S3.jpeg]

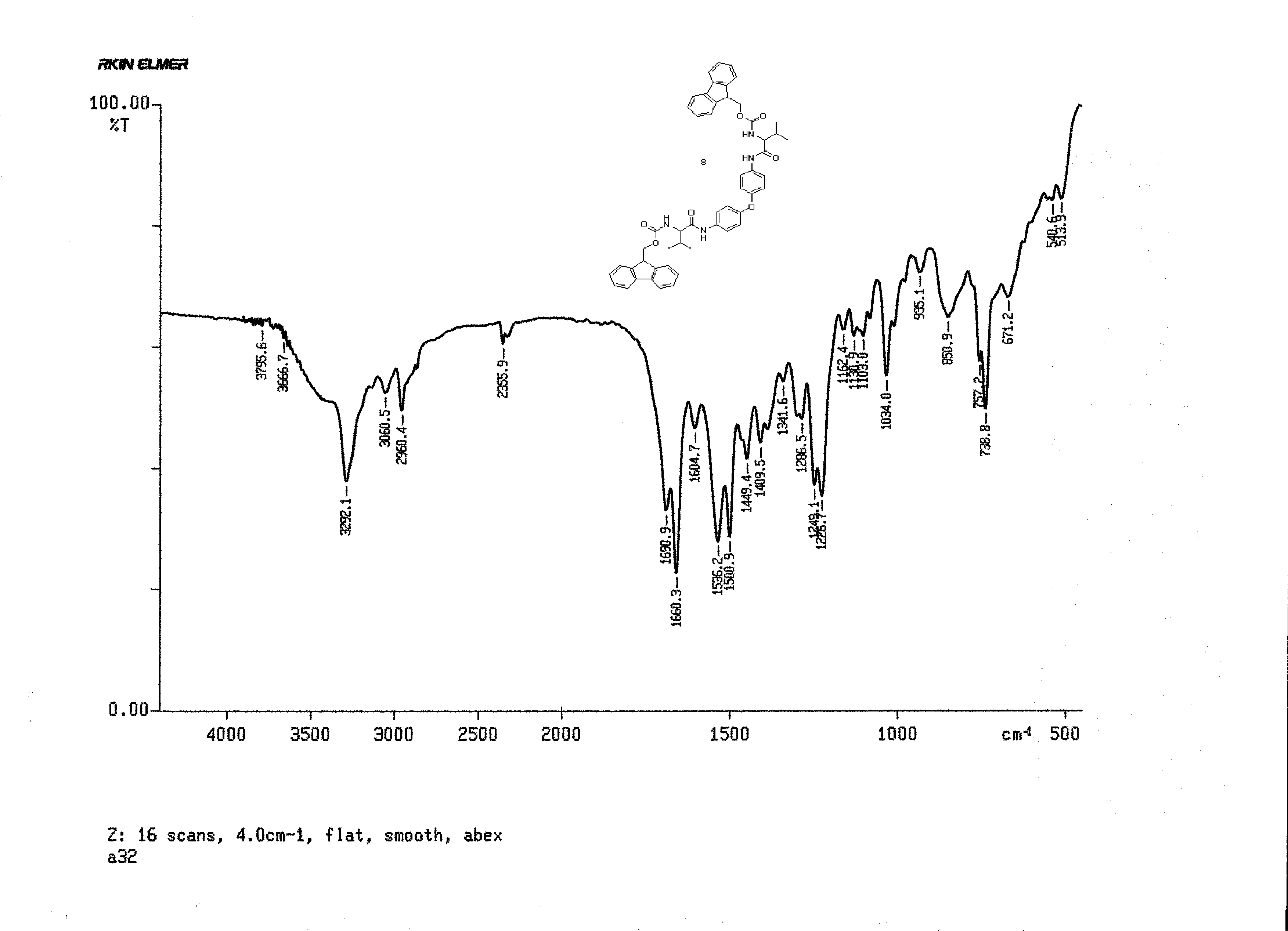

Supplement: Additional file 4 — FT-IR spectra of compound 6. [file 1752-153X-6-128-S4.tiff]

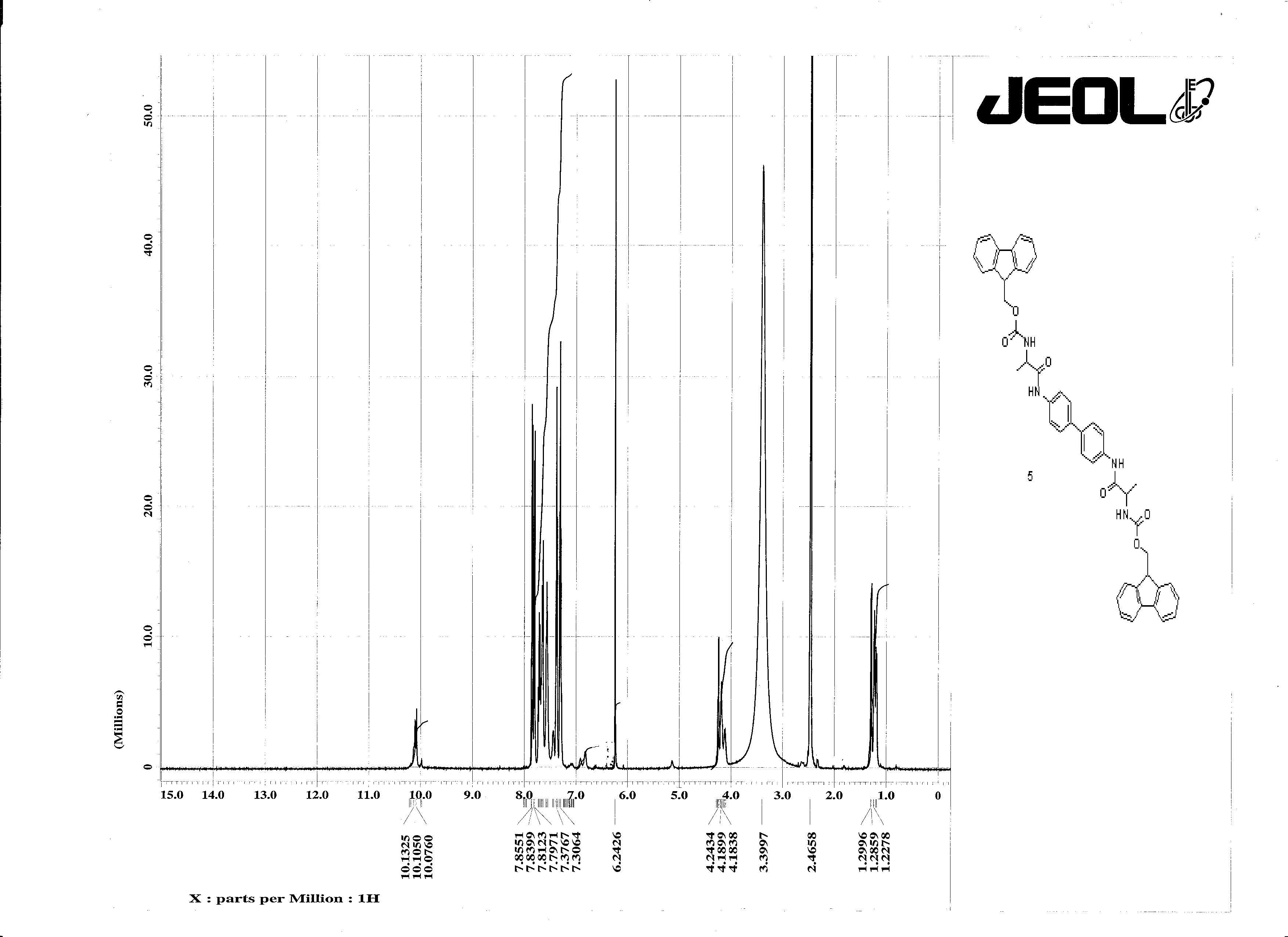

Supplement: Additional file 5 — 1H NMR spectra of compound of compound 6. [file 1752-153X-6-128-S5.tiff]

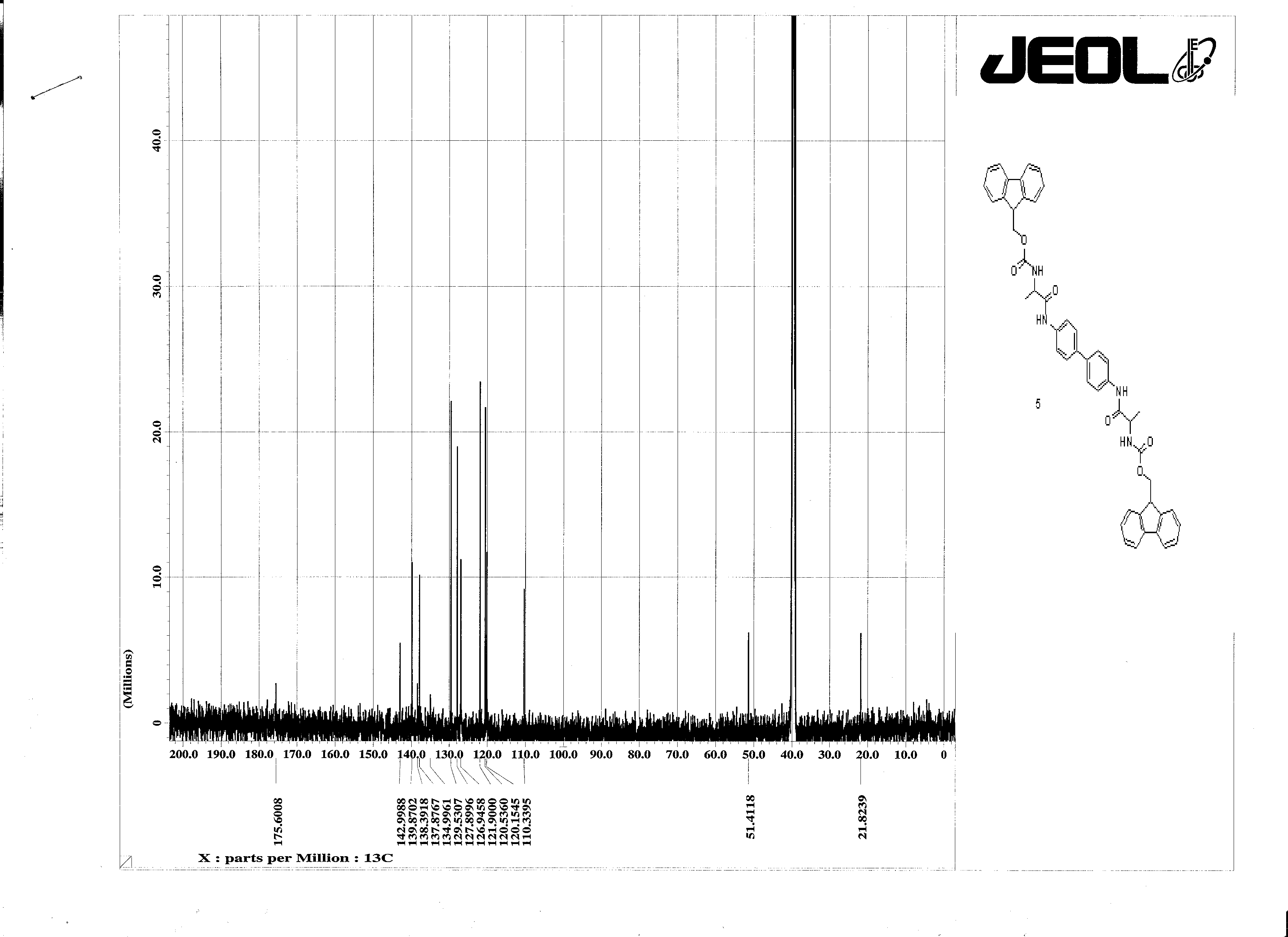

Supplement: Additional file 6 — 13C NMR spectra of compound of compound 6. [file 1752-153X-6-128-S6.tiff]

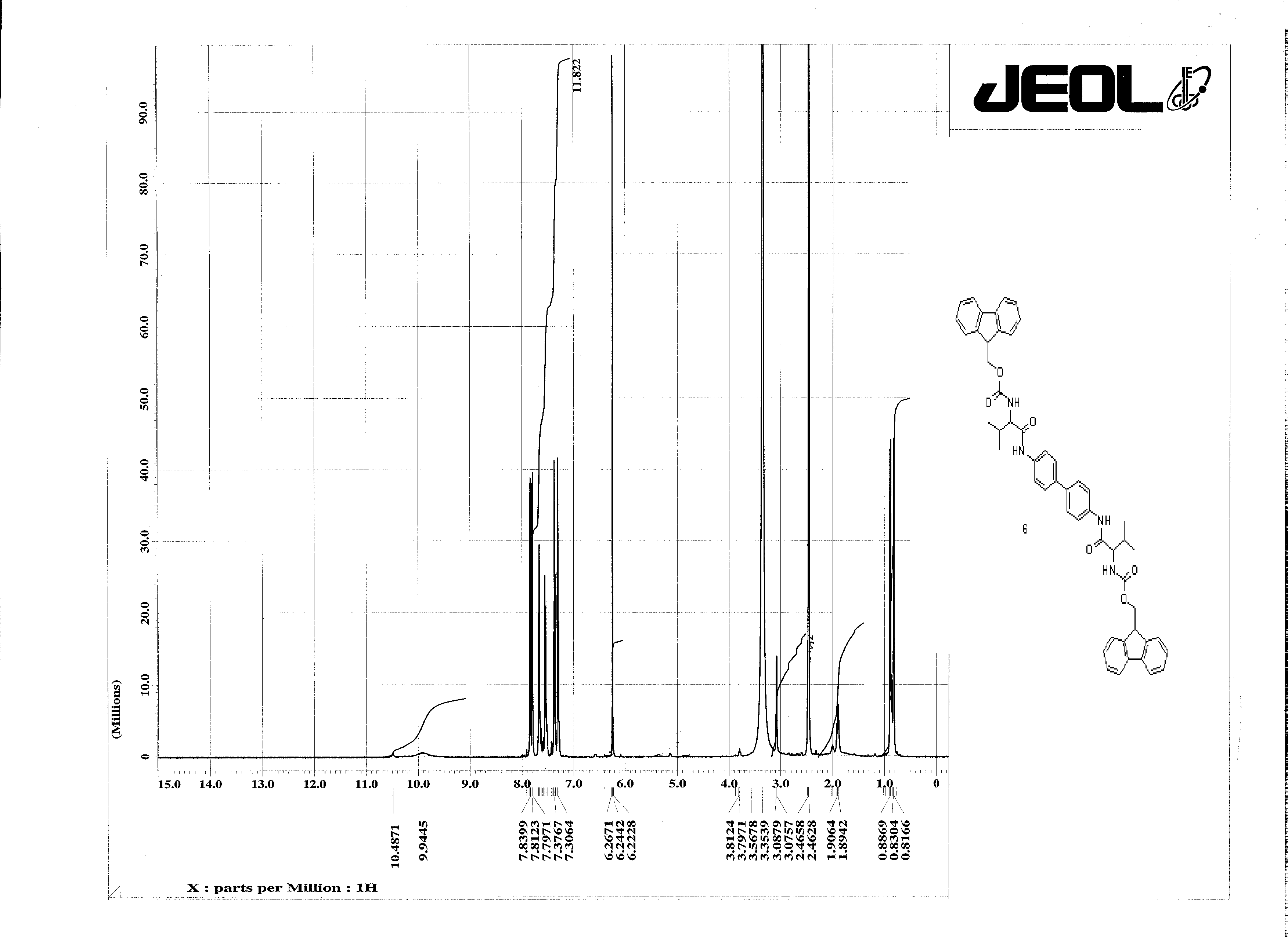

Supplement: Additional file 7 — FT-IR spectra of compound 7. [file 1752-153X-6-128-S7.tiff]

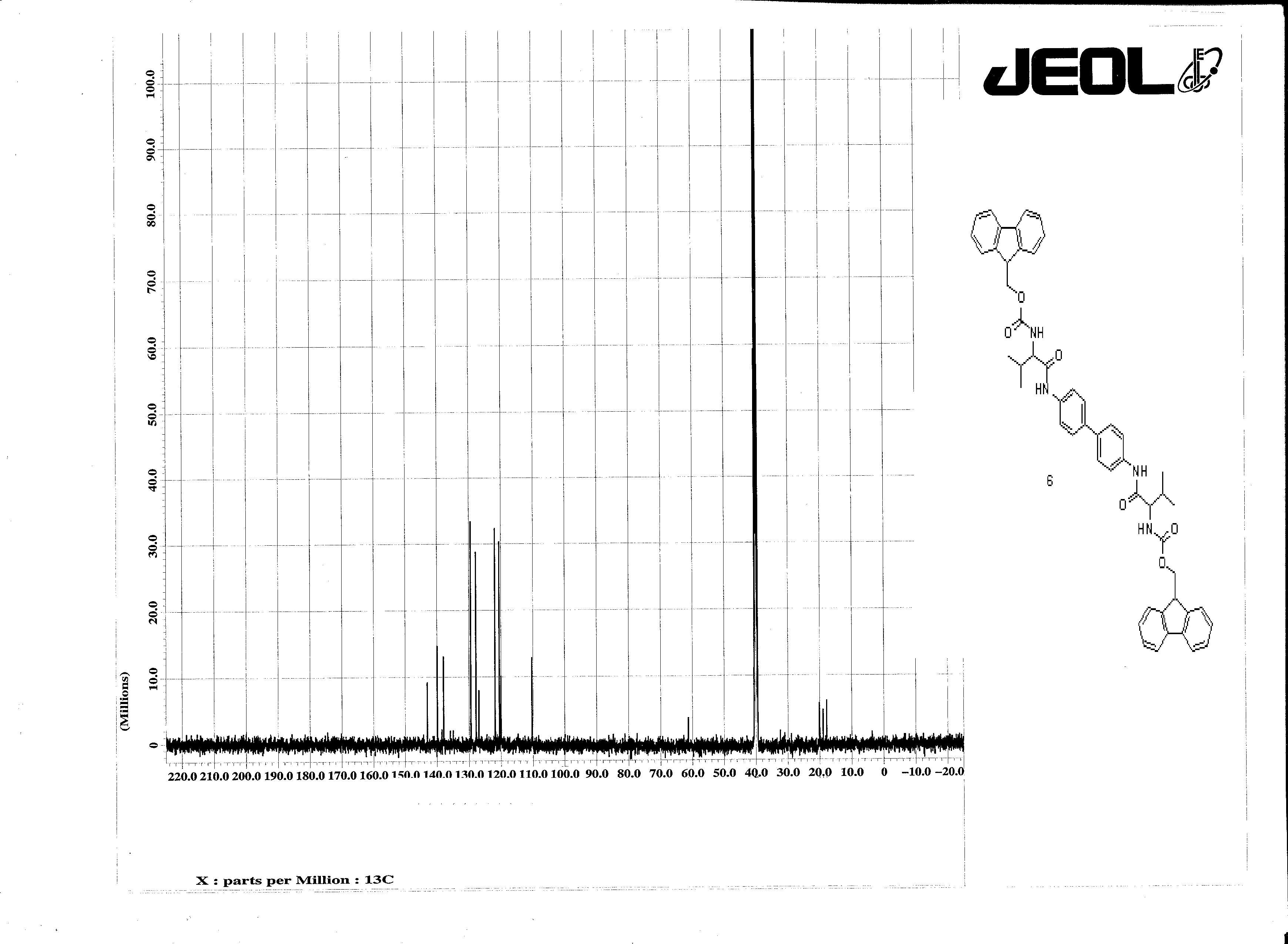

Supplement: Additional file 8 — 1H NMR spectra of compound of compound 7. [file 1752-153X-6-128-S8.tiff]

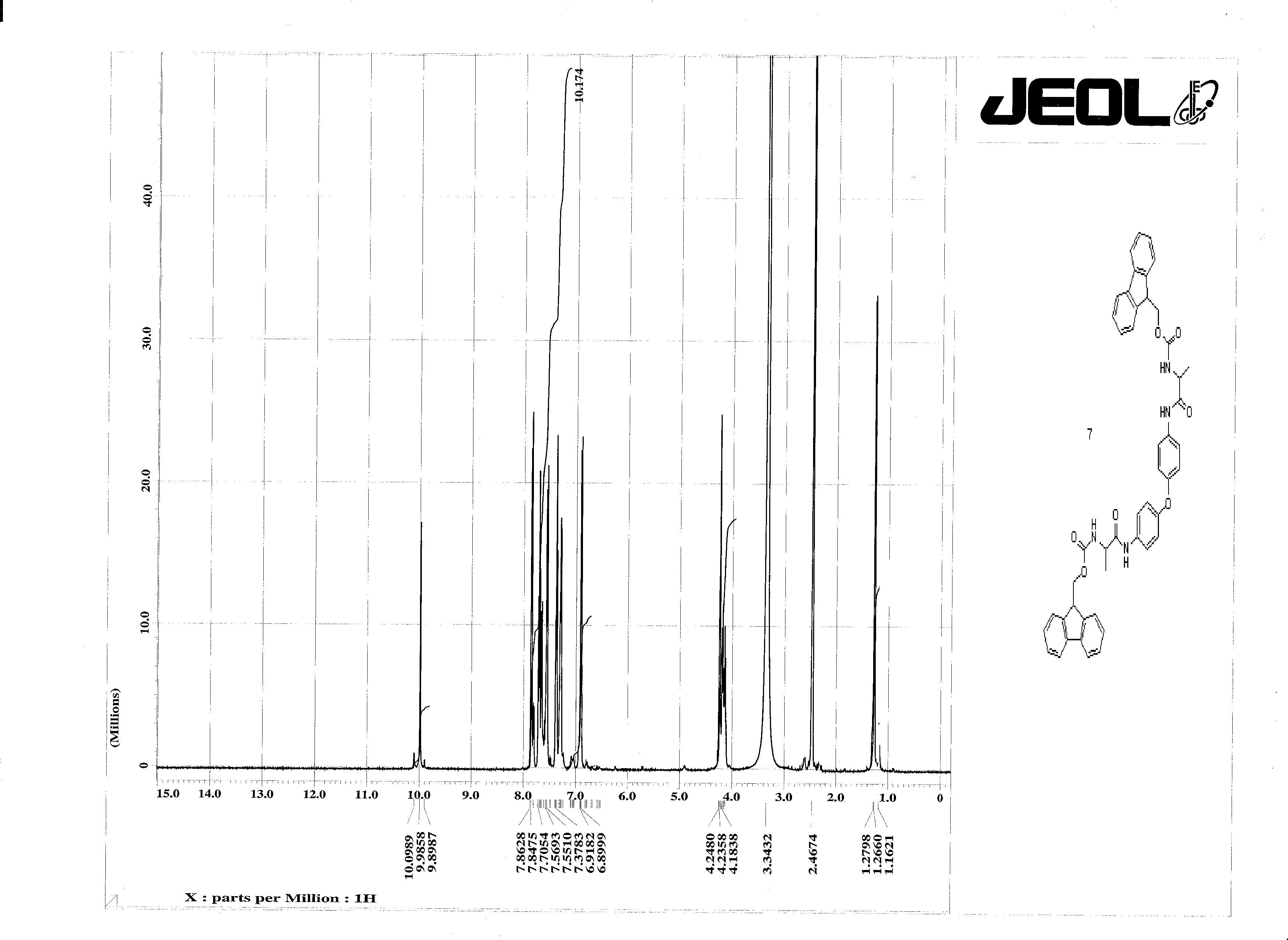

Supplement: Additional file 9 — 13C NMR spectra of compound of compound 7. [file 1752-153X-6-128-S9.tiff]

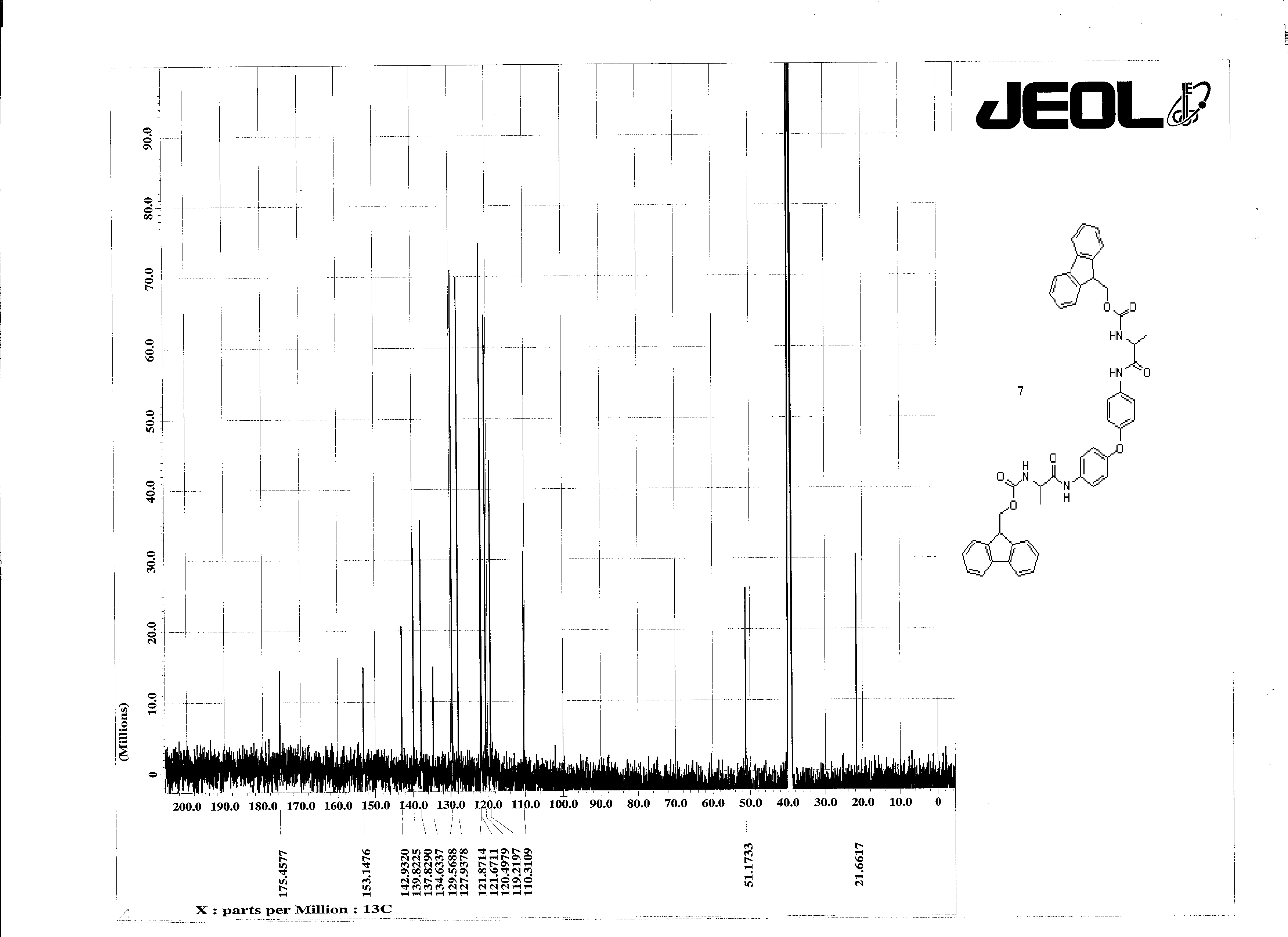

Supplement: Additional file 10 — FT-IR of compound 8. [file 1752-153X-6-128-S10.tiff]

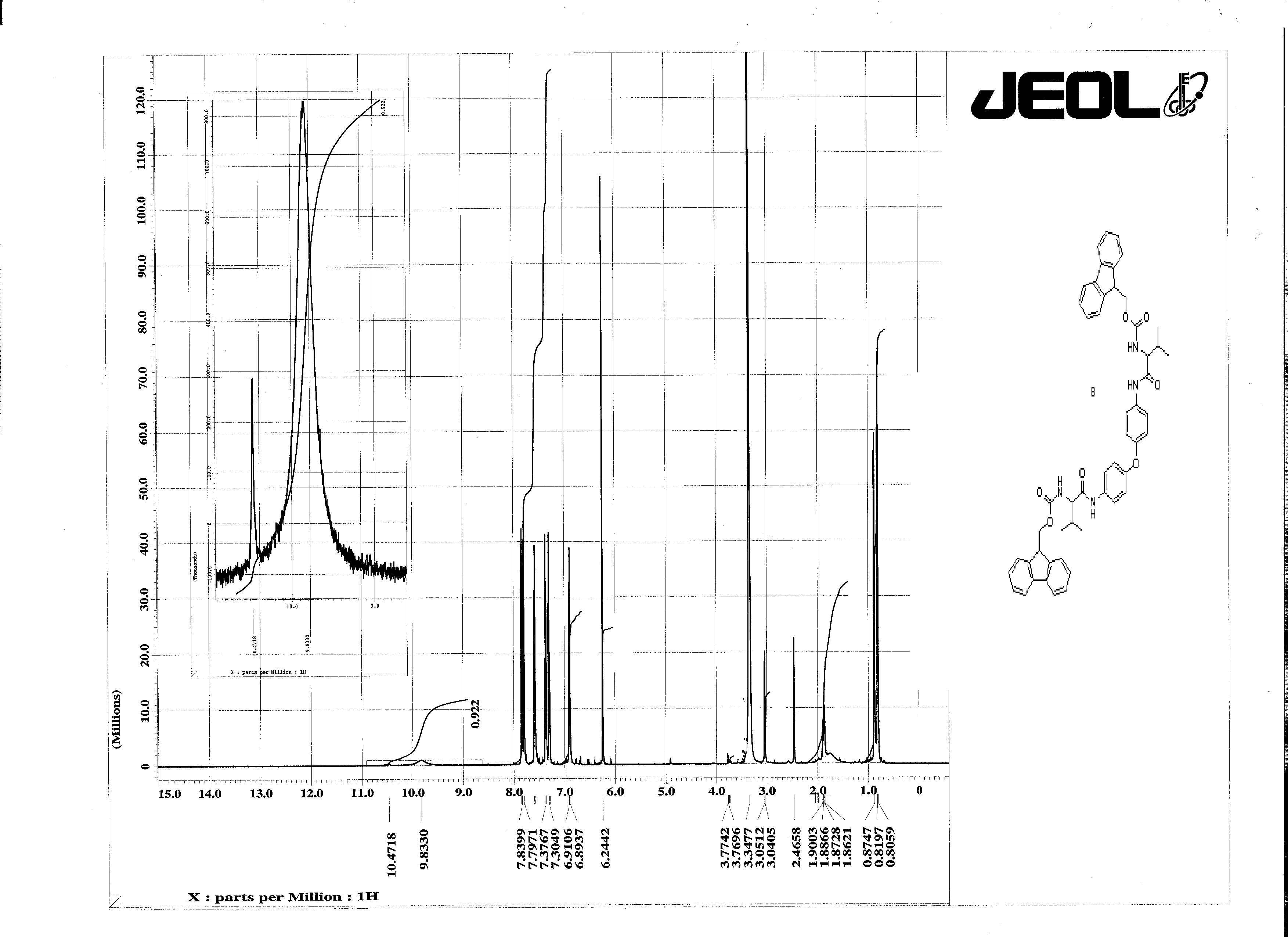

Supplement: Additional file 11 — 1H NMR spectra of compound of compound 8. [file 1752-153X-6-128-S11.tiff]

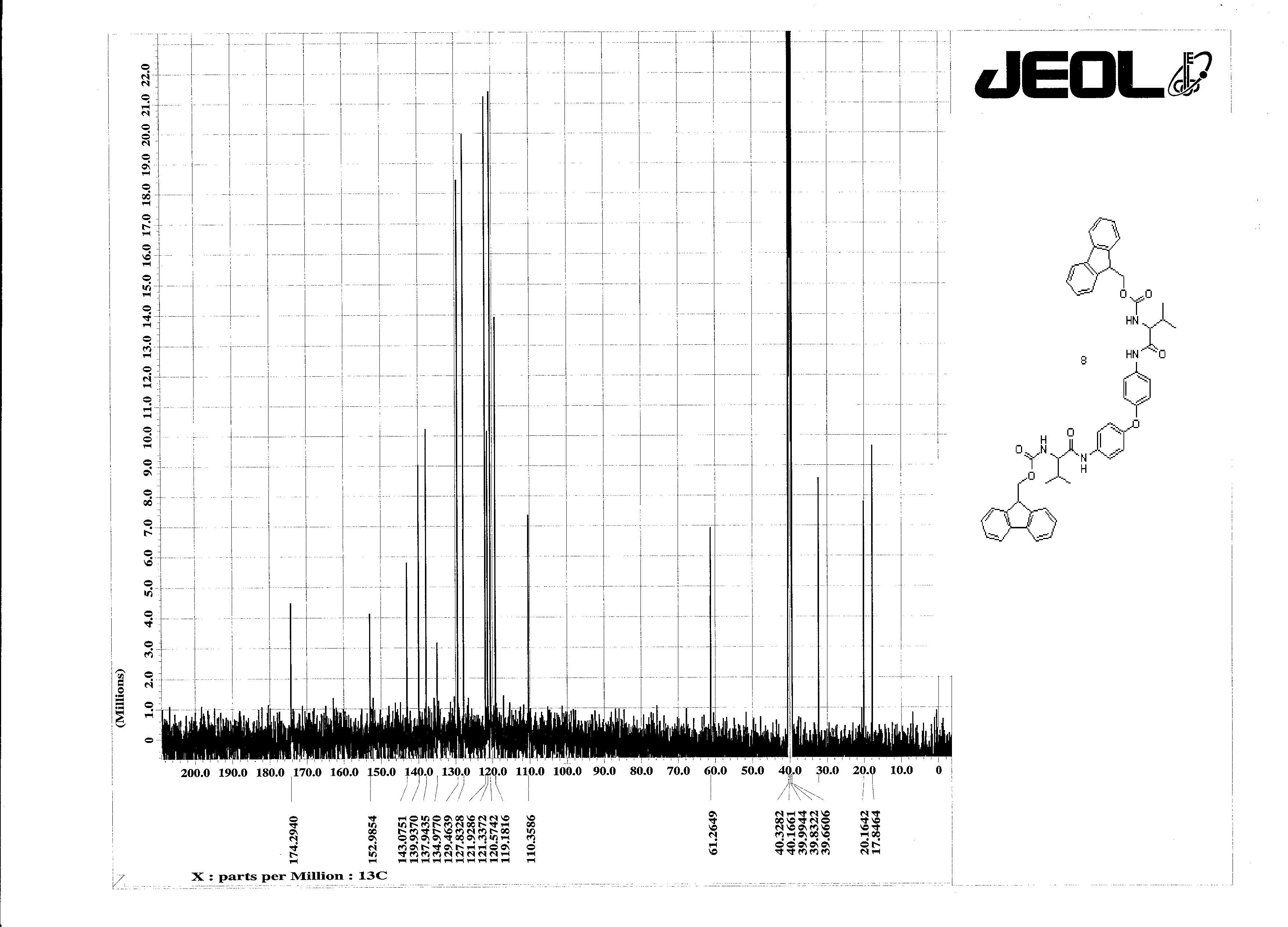

Supplement: Additional file 12 — 13C NMR spectra of compound of compound 8. [file 1752-153X-6-128-S12.tiff]
